# Interim Evaluation of the Secondary 2 Program of Project P.A.T.H.S.: Insights Based on the Experimental Implementation Phase

**DOI:** 10.1100/tsw.2008.22

**Published:** 2008-01-14

**Authors:** Daniel T. L. Shek, Rachel C. F. Sun, Andrew M. H. Siu

**Affiliations:** ^1^ Quality of Life Centre, Hong Kong Institute of Asia-Pacific Studies, The Chinese University of Hong Kong, Hong Kong; ^2^ Kiang Wu Nursing College of Macau, Macau; ^3^ Social Welfare Practice and Research Centre, The Chinese University of Hong Kong, Hong Kong; ^4^ Department of Rehabilitation Science, The Hong Kong Polytechnic University, Hong Kong

**Keywords:** interim evaluation, positive youth development, Chinese adolescents

## Abstract

An interim evaluation was conducted to understand the implementation quality of the Tier 1 Program (Secondary 2 Curriculum) of the Project P.A.T.H.S. (Positive Adolescent Training through Holistic Social Programs) in the Experimental Implementation Phase. Twenty-five schools were randomly selected to participate in personal and/or telephone interviews to provide information on the implementation details of the program and perceived attributes of the worker-support scheme (“Co-Walker Scheme”). Results showed that a majority of the workers perceived that the students had positive responses to the program and the program was helpful to the students. They also identified several good aspects in the program and the Co-Walker Scheme, albeit expressing some negative comments on the program design and difficulties in the implementation process. In conjunction with other findings reported previously, the present findings suggest that the Tier 1 Program is well received by different stakeholders and it promotes the positive development of secondary school students in Hong Kong.

## INTRODUCTION

Positive Adolescent Training through Holistic Social Programmes (P.A.T.H.S.) is a positive youth development program financially supported by The Hong Kong Jockey Club Charities Trust to promote holistic adolescent development in Hong Kong[1,2]. There are two implementation phases in this project – Experimental Implementation Phase (2005/06 to 2007/08 school years) and Full Implementation Phase (2006/07 to 2009/2010 school years). For the Tier 1 Program, the research team developed a set of curriculum manuals[1,2,3] that includes materials based on 15 positive youth development constructs identified from the existing successful positive youth development programs[4], and had taken reference with relevant research findings and existing programs in both local and foreign contexts. To provide support to colleagues who implement the Tier 1 Program, the research team had launched a "Co-Walker Scheme" since December 2006, which aimed to provide ongoing support and guidance to the participating schools.

Although papers documenting the positive program effects of the Tier 1 Program of P.A.T.H.S. have been published[5,6,7,8,9,10,11,12,13,14,15,16,17], they are limited to the Secondary 1 curriculum. As mentioned by Shek et al.[18], there are several developmental characteristics of Secondary 2 students. These include adjustment to puberty, cognitive maturation, rapid expansion of social circle, increased stress, encountering higher levels of social expectations, and gradual detachment from the family. Furthermore, they pointed out that compared with Secondary 1 students, Secondary 2 students have poorer psychological well-being and adaptation skills, and they perceived family functioning to be poorer and parental control to be looser. With such characteristics in Secondary 2 students, it is important to ask whether their reactions to positive youth development programs would be different from those of Secondary 1 students. Therefore, understanding the reactions of the students and the program implementers in the process is important.

Similar to the evaluation of the Secondary 1 Curriculum in the first year of the Experimental Implementation Phase[5] and Full Implementation Phase[6], an interim evaluation of the Secondary 2 Curriculum was carried out to examine the views of the workers regarding the implementation of the Tier 1 Program, based on a random sample of schools joining the second year of the Experimental Implementation Phase in the 2006/07 school year. As there are no empirical studies on process evaluation, it is argued that more work on process evaluation should be done[19,20,21,22].

## METHODS

### Participants

There were 49 schools that joined the Secondary 2 curriculum of the Project P.A.T.H.S. in the Experimental Implementation Phase in the 2006/07 academic year. Among these schools, 21 adopted the 20-h full program that involves 40 teaching units and 28 adopted the 10-h core program that involves 20 teaching units. Among these participating schools, 14 schools that joined the full program and 11 schools that joined the core program were randomly selected to participate in this study. In these selected schools, the instructors of the program (either school teachers or social workers) were invited to participate in face-to-face interviews on a voluntary basis during a school visit. If the respondents were not available for interviews during the school visit, they were invited to participate in telephone interviews or to complete self-administered questionnaires and return via e-mail or fax. Among these 25 schools, 15 took part in face-to-face interviews, six participated in telephone interviews, and four completed self-administered questionnaires. The respondents included 23 teachers and nine social workers. With reference to Principle 2 in the implementation of qualitative evaluation research proposed by Shek et al.[23], the number of schools that participated in this research can be regarded as respectable, as about half of the participating schools of the project in the second year of the Experimental Implementation Phase joined the interviews. Moreover, because the schools were randomly selected, the generalizability of the findings could be enhanced.

### Procedures

The procedures for data collection are systematically described below, and this satisfied Principle 3 in the procedures of a qualitative research[23]. The data collection was conducted between January and April 2007. As the Experimental Implementation Phase took place from September 2006 to August 2007, January to April 2007 can be regarded as the midpoint of the implementation process. While the presence of interviewers may affect the responses of the respondents (e.g., social desirability effect) in face-to-face interviews, it is unlikely to happen because the interviewers were the co-walkers of the interviewed schools with which a friendly rapport and mutual trust had been built. Also, the major advantage of face-to-face interviews is having the opportunity to clarify any doubts instantly. On the other hand, while telephone interviews and self-administered questionnaires have the problems of psychological distance and inability to observe the nonverbal cues of the respondents, their major advantage is efficiency in collecting the data within the time limit. In addition, follow-up calls could be arranged if there was a need to clarify the responses of the respondents. Therefore, these data collection methods have complemented each other.

A self-constructed semi-structured interview guide with five closed-ended questions and seven open-ended questions was used to collect information on the program implementation process. The open-ended questions were:

- Question 1: What are the responses of the students to this program?
- Question 2: Do you think this program is beneficial to the students? If yes, what are the benefits?
- Question 3: What are the good aspects of the program?
- Question 4: Which areas of the program require improvement?
- Question 5: Have you encountered any difficulties during the program implementation process? If yes, what problems have you encountered?
- Question 6: What are your perceptions of the “Co-Walker Scheme”?
- Question 7: Do you have other opinions?

Informed consent was obtained from the respondents and they participated in the study in a voluntary manner. The interviews were conducted by six co-walkers, who were a colleague having a doctoral degree and five registered social workers with substantial working experience. After each interview, the interviewers were required to fill in the questionnaires. The completed questionnaires were then transcribed and analyzed.

## Data Analyses

For the quantitative data (close-ended questions), frequencies and percentages of responses were calculated. The qualitative data (open-ended questions) were analyzed using general qualitative analyses techniques[24]. There were three steps in the process. First, relevant raw codes were developed for words, phrases, and/or sentences that formed meaningful units at the raw responses level. Second, the codes were further combined to reflect higher-order attributes at the category of codes level. Third, the categories of codes were further analyzed to reveal the broader themes at the thematic level. For example, the response to “involved in video watching” at the raw response level could be subsumed under the category of “high student involvement”, which could be further subsumed under the broad theme of “positive responses” of the students to the program (see Table 2). Following the principles of qualitative analyses[23], the raw data and categorized data were kept by a systematic filing system in order to ensure that the findings are auditable.

The qualitative data were coded and categorized by two trained research assistants, of which one has a Bachelor Degree of Psychology and another has a Master Degree of Social Work, which were further checked by the second author. In the present analyses, as the researchers designed the program in the Project P.A.T.H.S., they were conscious of their own biases and expectation on the program to be effective. In addition, in order to minimize the possible biases involved, both intra- and inter-rater reliability on the coding was calculated. For intrarater reliability, a research assistant and the second author individually coded 20 randomly selected responses for each question. For inter-rater reliability, a research assistant with a Master Degree and another research assistant who is a registered social worker, who was not involved in the data collection and analyses, coded 20 randomly selected responses for each question without knowing the original codes given at the end of the scoring process with reference to the codes finalized by the first author. This met Principles 4–9 of conducting a qualitative study advocated by Shek et al.[23].

## RESULTS

### Responses of the Students to the Program

About 92% of the respondents reported that the students were involved and liked the program (Table 1) and most of the perceived student responses were positive (64.9%) (Table 2). The intrarater agreement percentages were 100 and 100%, and the inter-rater agreement percentages were 80 and 95%, respectively, for responses reported in Table 2.

**TABLE 1**
**Instructors' Ratings of the Secondary 2 Curriculum (N and Percentage)**

1. Perceived degree of student involvement

<table>
<tr><th></th><th>Totally Not Involved</th><th>Not Involved</th><th>Involved</th><th>Totally Involved</th><th>No Response</th><th>Total</th></tr>
<tr><td>N</td><td>0</td><td>2</td><td>22</td><td>1</td><td>0</td><td>25</td></tr>
<tr><td>%</td><td colspan="2">8</td><td colspan="2">92</td><td>0</td><td>100</td></tr>
</table>

2. Perceived degree of students' liking of the Secondary 2 Curriculum

<table>
<tr><th></th><th>Strongly Dislike</th><th>Dislike</th><th>Like</th><th>Strongly Like</th><th>No Response</th><th>Total</th></tr>
<tr><td>N</td><td>0</td><td>2</td><td>23</td><td>0</td><td>0</td><td>25</td></tr>
<tr><td>%</td><td colspan="2">8</td><td colspan="2">92</td><td>0</td><td>100</td></tr>
</table>

3. Perceived degree of helpfulness of the Secondary 2 Curriculum

<table>
<tr><th></th><th>Unhelpful</th><th>Not Very Helpful</th><th>Slightly Helpful</th><th>Helpful</th><th>Very Helpful</th><th>No Response</th><th>Total</th></tr>
<tr><td>N</td><td>0</td><td>1</td><td>11</td><td>11</td><td>2</td><td>0</td><td>25</td></tr>
<tr><td>%</td><td colspan="2">4</td><td colspan="3">96</td><td>0</td><td>100</td></tr>
</table>

4. Perceived degree of workers' liking of the Secondary 2 Curriculum

<table>
<tr><th></th><th>Strongly Dislike</th><th>Dislike</th><th>Like</th><th>Strongly Like</th><th>No Response</th><th>Total</th></tr>
<tr><td>N</td><td>0</td><td>0</td><td>15</td><td>3</td><td>7</td><td>25</td></tr>
<tr><td>%</td><td colspan="2">0</td><td colspan="2">72</td><td>28</td><td>100</td></tr>
</table>

5. Perceived degree of workers' overall satisfaction of the Secondary 2 Curriculum

| | Very Dissatisfied | Dissatisfied | Slightly Dissatisfied | Slightly Satisfied | Satisfied | Very Satisfied | No Response | Total |
|---|---|---|---|---|---|---|---|---|
| N | 0 | 0 | 2 | 7 | 16 | 0 | 0 | 25 |
| % | | 8 | | | 92 | | 0 | 100 |

**TABLE 2**
**Workers' Perceptions of the Responses of the Students to the Secondary 2 Curriculum**

| | **Total Count (%)** |
|---|---|
| Positive Responses | 50 (64.9%) |
| High student involvement (e.g., "involved in video watching/games", "active to voice out opinions", "willing to discuss") (n = 23) | |
| Welcomed by students (e.g., "liked the topics", "looking forward to attending the lessons") (n = 7) | |
| High student interest (e.g., "students were interested", "liked the PowerPoint", "liked flash game") (n = 8) | |
| Other responses (e.g., "positive responses", "relaxed", "open", "students could learn some important skills") (n = 12) | |
| Neutral Responses | 1 (1.3%) |
| "It depends on the nature of students" (n = 1) | |
| Negative Responses | 26 (33.8%) |
| Low student involvement (e.g., "not attentive", "students having low motivation participated less", "passive to share") (n = 8) | |
| Feeling bored (e.g., "students were bored by some topics", "students considered the program was too simple/childish/not creative/repeated") (n = 8) | |
| Other responses (e.g., "resistant"; "students could not grasp the topics", "disliked worksheets", "diminishing student motivation") (n = 10) | |
| Total | 77 (100%) |

## Perceived Benefits of the Program to the Students

About 96% of the respondents regarded the Tier 1 Program of the Secondary 2 Curriculum as helpful to the students (Table 1) and most of responses presented in Table 3 were positive in nature. The intrarater agreement percentages were 100 and 80%, and the inter-rater agreement percentages were 85 and 90%, respectively.

## Positive Aspects of the Program and Areas that Require Improvement

As shown in Table 4, there were 72 meaningful units regarding the positive aspects of the program. The intrarater agreement percentages were 100 and 95%, and the inter-rater agreement percentages were 95 and 90%, respectively. On the other hand, there were 51 meaningful units concerning the aspects of the program requiring improvement (Table 5). About half of the responses indicated the "curriculum content and activity design" needed to be modified (51.0%), while some responses suggested improving the "teaching manuals and resources" (17.6%) and adjusting the "implementation time" (11.8%). The intrarater agreement percentages were 100 and 100%, and the inter-rater agreement percentages were 80 and 85%, respectively.

## Difficulties Encountered during Program Implementation

As shown in Table 6, there were 44 meaningful units indicating the workers' perception of the difficulties encountered during program implementation. Among them, most of the responses were related to difficulties in "administration and coordination" (31.8%), which was followed by the perceived difficulties in "time management" (13.6%) and "heavy workload" (13.6%), then in handling "students' responses" (11.4%) and the "curriculum content and activity design" (9.1%). The intrarater agreement percentages were 100 and 85%, and the inter-rater agreement percentages were 90 and 85%, respectively.

**TABLE 3**
**Workers' Perceptions of the Benefits of the Secondary 2 Curriculum**

| | **Total Count (%)** |
|---|---|
| Undecided | 8 (17.8%) |
| Facilitating Students' Holistic Development | 6 (13.3%) |
| "The content is broad/related to daily life" (n = 4) | |
| "It facilitates students' growth/development" (n = 2) | |
| Building Up Interpersonal Relationships | 7 (15.6%) |
| "Students learn how to choose/get along with friends" (n = 2) | |
| "It enables students to understand/get along with others" (n = 4) | |
| "It enables students to get along with their parents" (n = 1) | |
| Strengthening Students' Behavioral and Cognitive Competence | 8 (17.8%) |
| "It enables students' thinking skills" (n = 5) | |
| "It enables students' problem-solving/goal-setting/presentation skills" (n = 3) | |
| Enhancing Students' Self-Development | 9 (20.0%) |
| "It enables students to reflect on one's personality/values/developmental issues" (n = 6) | |
| "It enables students to have more self-understanding/self-growth" (n = 3) | |
| Facilitating Students' Learning | 7 (15.6%) |
| "Students had improvement in Secondary 2, when compared with their Secondary 1" (n = 2) | |
| "Students could tell what they had learned from the lessons" (n = 3) | |
| "It stimulates students' learning" (n = 2) | |
| Total | 45 (100%) |

## Perceptions of the "Co-Walker Scheme"

For the workers' perceptions of the "Co-Walker Scheme" (Table 7), about 60% responses were positive and 37.5% were "none/no comment/undecided". The intrarater agreement percentages were 100 and 90%, and the inter-rater agreement percentages were 100 and 100%, respectively.

## Other Opinions of the Project

About 72 and 92% of the respondents showed that they liked the program and felt satisfied, respectively (Table 1). The responses to the open-ended question of other opinions of the project were shown in Table 8. The intrarater agreement percentages were 100 and 80%, and the inter-rater agreement percentages were 85 and 95%, respectively.

## DISCUSSION

Several phenomena could be highlighted from the present study. First, the respondents felt that most of the students had positive responses to the program, as shown in the findings reported in Tables 1–4. These findings are similar to the interim evaluation findings on the Tier 1 Program (Secondary 1 Curriculum) in the Full Implementation Phase (2006/07 school year)[6], and further supported the findings arising from the objective outcome evaluation[7,8], subjective outcome evaluation[9,10,11,12,13], qualitative evaluation based on student focus group[14] and students' weekly diaries[15], and process evaluation in

**TABLE 4**
**Workers' Perceptions of the Positive Aspects of the Secondary 2 Curriculum**

| | **Total Count (%)** |
|---|---|
| None | 1 (1.4%) |
| Teaching Manuals and Resources | 28 (38.9%) |
| Diverse and good teaching aids (e.g., "it has multimedia resources", "they make students more involved", "teaching aids are better than those in Secondary 1 Curriculum") (n = 16) | |
| Sufficient teaching resources (e.g., "comprehensive teaching materials", "rich resources") (n = 7) | |
| Clear teaching plans (e.g., "teaching plans are clearly presented", "teaching plans are detailed") (n = 3) | |
| Other responses (e.g., "teaching materials/CD-ROMs are user friendly") (n = 2) | |
| Curriculum Content and Activity Design | 31 (43.1%) |
| Good curriculum content (e.g., "diverse topics", "rich content", "comprehensive coverage", "Secondary 2 Curriculum content is more in-depth/concentrated than Secondary 1 Curriculum content", "structural", "close to daily life", "it meets students' needs") (n = 18) | |
| Good activity design (e.g., "there are small group activities", "the activity design is active/diverse/rich/interesting") (n = 12) | |
| Other responses (e.g., "teachers can handle it") (n = 1) | |
| Philosophy | 6 (8.3%) |
| Good positive youth development constructs (e.g., "the constructs are important to students' growth", "they are comprehensive") (n = 3) | |
| Good theoretical basis (e.g., "good theoretical framework") (n = 3) | |
| Other Responses | 6 (8.3%) |
| Beneficial to students and teacher-student relationship (n = 2) | |
| Having sufficient resources (n = 2) | |
| Having teacher support (n = 1) | |
| Having support from the project (n = 1) | |
| Total | 72 (100%) |

terms of observation[16] and interim evaluation[5] of the Secondary 1 Curriculum in the Experimental Implementation Phase (2005/06 school year) that both the students and workers had positive perceptions of the program, and the program was beneficial to the students.

Besides, the respondents also pointed out several aspects of the program that require improvement and difficulties encountered in the implementation process (Tables 5, 6, and 8). Similar to previous findings[5,6], about half of the responses referred to the curriculum content and activity design requiring modifications. As indicated in the previous papers[17,25], the experiential learning approach underlining the Tier 1 Program of Project P.A.T.H.S. requires the collaboration between teachers and students that may not be entirely consistent with the traditional Chinese way of education (emphasis on didactic learning) and authority figure of the teachers[26].

In addition, similar to previous findings[5,6], nearly one-third of the responses reported having difficulties in administration, and coordination and time management. Although it was the second year of program delivery in the Experimental Implementation Phase, it is possible that some schools deployed much manpower to run the Secondary 1 and 2 programs. Furthermore, for cases where the program was incorporated into the formal curriculum, assessment and covering the syllabus are stressors for the program implementers. Although there could be different interpretations of the above findings, these

**TABLE 5**
**Workers' Perceptions of the Aspects of the Secondary 2 Curriculum that Require Improvement**

| | **Total Count (%)** |
|---|---|
| None | 2 (3.9%) |
| Curriculum Content and Activity Design | 26 (51.0%) |
| "The activities are too many/too complex/too shallow/cannot arouse students' interests" (n = 7) | |
| "Provide alternatives to the activities/add more activities" (n = 2) | |
| "The teaching units are loosely linked" (n = 2) | |
| "Some topics are repeated/do not match with the construct" (n = 3) | |
| "Remove the topics that students have less exposure/not up to date/less close to daily life" (n = 6) | |
| Other responses (e.g., "some activity formats are similar", "use students' as positive role models") (n = 6) | |
| Teaching Manuals and Resources | 9 (17.6%) |
| "Add more videos/PowerPoint" (n = 5) | |
| "Worksheets are too many/difficult" (n = 2) | |
| Other responses (e.g., "no English version", "to have student handbooks") (n = 2) | |
| Implementation Time | 6 (11.8%) |
| "There is insufficient time for running the activities" (n = 3) | |
| "Time is pressing/often overruns" (n = 2) | |
| Other responses, (e.g., "continuity of the program is affected because the lessons were not carried out persistently") (n = 1) | |
| Other Responses | 8 (15.7%) |
| "It is difficult to have small group discussion in a large class" (n = 2) | |
| "Workers need to tailor make the program for students" (n = 2) | |
| "The long program makes it difficult to arrange classroom lessons/to follow-up" (n = 2) | |
| "To simplify the administrative work so as to have more time for preparation and teaching" (n = 1) | |
| "Teaching skills are needed" (n = 1) | |
| Total | 51 (100%) |

comments made by the respondents provide invaluable feedback on further improvement of the program, and the research team would seriously consider the comments collected and recorded. Also, it is noteworthy that the present findings provide an opportunity for the school or agency administrators and workers to reflect on how their administration and coordination, time management, teaching skills, and attitudes could be further improved.

Regarding the “Co-Walker Scheme”, as it had just been launched for 1–4 months at the time of data collection, it is understandable that about 38% of respondents had no comment or could not comment on it (Table 7). Nonetheless, a majority of the responses (60%) indicated that the scheme was good and welcomed by the workers.

Following Principle 10 of conducting a qualitative study[23], three possible alternative explanations were put forward for the present findings – demand characteristics, selection bias, and ideological biases of the researchers. However, these explanations are not likely because (1) the respondents were encouraged to voice their views without restriction and negative voices were, in fact, heard; (2) the schools were randomly selected; and (3) several safeguards were used to reduce biases in the data collection and analysis process, such as multiple colleagues involved to provide checking for individual biases, as well as intra- and inter-rater reliability checks carried out to safeguard consistency in coding.

**TABLE 6**
**Workers’ Perceptions of the Difficulties Encountered in Secondary 2 Curriculum Implementation**

| | **Total Count (%)** |
|---|---|
| None | 1 (2.3%) |
| Administration and Coordination | 14 (31.8%) |
| “It was difficult to arrange a time table” (n = 5) | |
| “It was difficult to arrange manpower/division of labor” (n = 4) | |
| “The cooperation between the school and social work agency/between teachers and social workers was not satisfactory” (n = 2) | |
| Other responses, (e. g., “not enough classrooms”, “need to get support from school principal and teachers”) (n = 3) | |
| Workload | 6 (13.6%) |
| “Teachers had to spend energy for preparation/workload became heavier” (n = 5) | |
| “It was hard for one teacher to teach a class” (n = 1) | |
| Time Management | 6 (13.6%) |
| “There was insufficient time to run the program” (n = 2) | |
| “Time was pressing, and often overran” (n = 3) | |
| “Time was wasted for setting up the audio-visual equipments” (n = 1) | |
| Curriculum Content and Activity Design | 4 (9.1%) |
| “The content was too much” (n = 2) | |
| “Some topics were not up to date/too conceptual and vivid, and thus difficult to handle” (n = 2) | |
| Students’ Responses | 5 (11.4%) |
| “Classroom disorder was a problem” (n = 2) | |
| “Students did the tasks poorly/resisted to have small group discussion” (n = 3) | |
| Other Responses | 8 (18.2%) |
| “The audio-visual system in school was poor/insufficient” (n = 3) | |
| “Teachers did not acquire adequate skills to run activities” (n = 3) | |
| “Social workers did not get acquainted with the students” (n = 1) | |
| “It was difficult to assess students’ learning” (n = 1) | |
| Total | 44 (100%) |

According to Principle 12 proposed by Shek et al.[23], several limitations of the study are described below. First, as each interview was brief and some data were collected through self-administrated

**TABLE 7**
**Workers' Perceptions of the "Co-Walker Scheme" (Secondary 2 Curriculum)**

| | **Total Count (%)** |
|---|---|
| None/No Comment/Undecided | 15 (37.5%) |
| Positive Responses | 24 (60.0%) |
| "It is fine/good/very good" (n = 9) | |
| "It can support schools" (n = 3) | |
| "It can strengthen communication" (n = 5) | |
| "It enables understanding to the needs of frontline workers" (n = 2) | |
| "The co-walkers/workers can know more about the program implementation in different schools" (n = 5) | |
| Neutral Responses | 0 (0%) |
| Negative Responses | 0 (0%) |
| Other Responses | 1 (2.5%) |
| "It needs to clearly define the role of co-walkers, so as to avoid expectation discrepancies" (n = 1) | |
| Total | 40 (100%) |

**TABLE 8**
**Other Opinions of the Project (Secondary 2 Curriculum)**

| | **Total Count (%)** |
|---|---|
| None | 14 (50.0%) |
| Curriculum Content and Activity Design | 5 (17.9%) |
| "Having too many worksheets" (n = 1) | |
| "The curriculum content can be more challenging to students" (n = 1) | |
| "Include more discussion/reflection/soundtracks and videos" (n = 3) | |
| School Administration and Co-ordination | 3 (10.7%) |
| "If teachers are in charge of the program, it is hoped that the school can receive financial support" (n = 1) | |
| "Some teachers do not understand the rationales of the project" (n = 1) | |
| "It is difficult for school teachers to arrange a time for sharing" (n = 1) | |
| Other Comments and Suggestions | 6 (21.4%) |
| "Too much training for workers" (n = 1) | |
| "The research team can print growth puzzles and worksheets for students" (n = 1) | |
| "To give more instructions to teachers" (n = 1) | |
| "Hoping to have such a structural curriculum for Secondary 4 and 5 levels) (n = 1) | |
| "To set up a resource bank in the web for sharing teaching materials/for understanding the implementation in other schools" (n = 2) | |
| Total | 28 (100%) |

questionnaires, the researchers were not able to collect in-depth information and construct "thick descriptions". Therefore, in-depth individual or focus group interviews would be helpful to further understand the subjective experiences of the program implementers and participants. Second, this interim evaluation was a kind of process evaluation of a program with the program implementers only; therefore, another qualitative study that utilized students' weekly diaries to collect students' perceptions of the Secondary 2 Program was also conducted at the end of the program to further explore the program implementation quality. Despite these limitations, this study in the second year of the Experimental Implementation Phase provides interim evaluation findings that contribute to the Chinese literature on positive youth development programs. It also supports the positive attributes of the Project P.A.T.H.S. and its effectiveness in promoting holistic youth development among Chinese adolescents in Hong Kong. Based on the principle of triangulation[27], evaluation findings based on different methods, different data, and different sources of information generally provide evidence for the effectiveness of the Tier 1 Program of the P.A.T.H.S. Project.

## ACKNOWLEDGMENTS

The preparation for this paper and the Project P.A.T.H.S. were financially supported by the Hong Kong Jockey Club Charities Trust.
